# Multimorbidity and cardiovascular prevention in primary care: a cohort study in New Zealand

**DOI:** 10.3399/BJGP.2025.0798

**Published:** 2026-07-28

**Authors:** Stina Mannheimer, Yeunhyang Catherine Choi, Katrina Poppe, Jingyuan Liang, Vanessa Selak, Allan Moffitt, Sue Wells

**Affiliations:** 1 University of Gothenburg Centre for Person-centred Care, Institute of Health and Care Sciences, University of Gothenburg, Gothenburg, Sweden; 2 Department of Epidemiology and Biostatistics, University of Auckland, Auckland, New Zealand; 3 Department of Medicine, University of Auckland, Auckland, New Zealand; 4 ProCare Health Limited, Auckland, New Zealand; 5 Head of Department of General Practice and Primary Healthcare, University of Auckland, Auckland, New Zealand

**Keywords:** cardiovascular preventive medication, general practice, multimorbidity, risk assessment

## Abstract

**Background:**

Multimorbidity adds complexity to cardiovascular disease (CVD) prevention. Clinical guidelines in Aotearoa New Zealand recommend combined blood pressure- and lipid-lowering therapy (‘dual therapy’) for most people at high (≥15%) 5-year CVD risk; recommend shared decision making for those at intermediate risk (5–14%); and do not generally recommend dual therapy for those at low risk (<5%).

**Aim:**

To examine the association between multimorbidity and dispensing of dual therapy across CVD risk groups.

**Design and setting:**

Cohort study of patients enrolled in a primary health organisation serving the Auckland region of northern New Zealand.

**Method:**

The study included 430 286 adults aged 30–79 years without prior CVD who were enrolled in ProCare in 2014. Multivariable Poisson regression was used to estimate relative risks (RRs) for receiving dual therapy within each CVD risk group. Two-year maintenance of dual therapy was compared by multimorbidity status.

**Results:**

Baseline dual therapy dispensing was 2.5%, 31%, and 40% in the low-, intermediate-, and high-risk groups, respectively. Multimorbidity was associated with a greater likelihood of receiving dual therapy across all risk groups (low risk: RR 2.90, 95% confidence interval (CI) = 2.77 to 3.03; intermediate risk: RR 1.35, 95% CI = 1.32 to 1.38; high risk: RR 1.15, 95% CI = 1.10 to 1.22). Among participants receiving dual therapy at baseline, maintenance was higher among those with multimorbidity in the low-risk group (88% versus 84%) and intermediate-risk group (90% versus 86%) (both *P*<0.001). In the high-risk group, maintenance was similarly high irrespective of multimorbidity (89% versus 88%; *P* = 0.412).

**Conclusion:**

Patients with multimorbidity were more likely to receive and maintain guideline-recommended CVD preventive therapy than those without multimorbidity. However, despite increasing use of dual therapy with increasing CVD risk, substantial evidence–practice gaps remained among patients at high CVD risk.

**How this fits in**

Current guidelines for cardiovascular disease (CVD) prevention are largely risk based, yet evidence on how multimorbidity affects their implementation remains limited. This large New Zealand cohort study examined the dispensing of dual therapy with blood pressure- and lipid-lowering medication across cardiovascular risk groups in people with, and without, multimorbidity. People with multimorbidity were more likely to receive dual therapy across risk strata; however, receipt of guideline-recommended treatment remained suboptimal among those at high risk of CVD.

## Introduction

Life expectancy is increasing in Aotearoa New Zealand but 20–30% of the additional years gained are lived with multimorbidity, defined as two or more long-term conditions.^1^ Globally, multimorbidity affects about two-thirds of people aged ≥65 years^2^ and accounts for 70% of GP visits.^3^ In New Zealand, multimorbidity becomes increasingly common with age and is more prevalent among Māori (indigenous people of New Zealand), Pacific peoples, and people living in socioeconomically deprived areas.^4^ Also, Māori, Pacific peoples,^5^ and South Asian peoples^6,7^ experience a disproportionately high burden of cardiovascular disease (CVD) compared with New Zealand Europeans.

New Zealand guidelines recommend combined blood pressure- and lipid-lowering therapy (‘dual therapy’) for most people at high (≥15%) 5-year CVD risk, support shared decision making for those at intermediate risk (5–14%),^7^ and do not generally recommend dual therapy for those at low risk (<5%).^5^ A cohort study of more than 2 million New Zealanders showed that CVD primary prevention therapy increased with risk, but only about half of people at high risk received recommended dual therapy.^8^

Although multimorbidity adds complexity to medication management,^9^ it remains unclear whether the use and maintenance of guideline-recommended CVD preventive therapy differ according to multimorbidity status. The authors therefore aimed to examine the association between multimorbidity and both dispensing and maintenance of dual therapy across CVD risk groups in New Zealand.

## Method

### Study design and population

This observational cohort study used data from patients registered with a general practice belonging to the ProCare Primary Health Organisation on 1 January 2014 (*n* = 623 475), with 2 years of follow-up. At the index date, ProCare comprised more than 170 general practices and served approximately 51% of Auckland’s population and 17% of the New Zealand population.

The study included adults aged 30–79 years without prior CVD or CVD risk-equivalent conditions. Demographic information, CVD risk factors, admissions to hospital, and medication dispensing records were linked using encrypted National Health Index (NHI) numbers,^10^ the unique healthcare identifier used throughout the New Zealand health system, enabling accurate patient-level linkage while preserving confidentiality. The datasets and their applicable date ranges are described in Supplementary Appendix S1).

Patients were excluded if they were aged <30 or >79 years (because the CVD risk equations used in this study apply to people aged 30–79 years), had sex recorded as ‘Other/Unknown’ (because the equations are sex specific), had missing New Zealand Deprivation Index data, or had prior CVD or conditions equivalent to high CVD risk (such as renal dialysis or transplantation, or congestive heart failure). Prior CVD was defined as admissions to hospital for atherosclerotic CVD, haemorrhagic stroke, cardiopulmonary transplant, at least three dispensings of antianginal agents in the previous 5 years, or relevant GP diagnoses (myocardial infarction, angina, cerebrovascular disease, aneurysm, or peripheral vascular disease). Congestive heart failure was defined as prior admission to hospital for this condition, repeated loop diuretic dispensing (at least three times in 5 years), or the dispensing of metolazone or an angiotensin receptor–neprilysin inhibitor in the previous 6 months. Primary care conditions were identified using a previously described text-based classification approach based on Read codes, Read terms, and free text.^11^ Hospital data were available from 1 January 1988.

#### Estimated cardiovascular disease risk

Key clinical variables required for the nationally implemented PREDICT^6^ CVD risk prediction model — including blood pressure, body mass index, and lipid measurements — were not consistently available across the administrative data sources used in this study. The authors therefore estimated 5-year risk of a first fatal or non-fatal CVD event using updated sex- and ethnicity-specific CVD risk prediction equations developed by J Liang and colleagues for routinely collected New Zealand health and dispensing data (unpublished personal communication with Jingyuan Liang, 10 September 2025). These equations were derived from a larger and more recent nationwide health-contact cohort than the original PREDICT model and represent an updated version of New Zealand’s validated national policy-focused CVD risk equations.^12^ The equations enabled internally consistent estimation of 5-year CVD risk across the entire cohort, and provided improved estimation of ethnicity-specific risk, particularly for Māori, Pacific, and Indian populations.

Variables included age, New Zealand Deprivation Index quintile, diabetes, prior hospital admission for atrial fibrillation, a smoking proxy, and dispensing of blood pressure-lowering, lipid-lowering, antithrombotic, or anticoagulant medications. Definitions of model variables and diagnostic codes for CVD outcomes are provided in Supplementary Appendices S2 and S3.

Five-year CVD risk was estimated for each participant at baseline (1 January 2014). When applied to the 2014 study cohort, the model demonstrated good discrimination (area under the receiver operating characteristic curve (AUC) = 0.815, 95% confidence interval [CI] = 0.811 to 0.818).

### Participant variables

#### Sociodemographic variables

Date of birth, sex, and self-identified ethnicity were obtained from the National Health Index collection. Age was grouped as 30–59 years, 60–69 years, or 70–79 years following sensitivity analyses. Ethnicity was prioritised according to modified New Zealand ethnicity data protocols^13^ as Māori, Pacific peoples, Indian, Chinese, European, and Other (including other Asian, Middle Eastern, Latin American, African, and other groups). Indian, the largest South Asian ethnic group in New Zealand, is recorded separately in national health data, whereas other South Asian ethnicities are included within the Other Asian classification.

Socioeconomic deprivation was measured using the 2013 New Zealand Deprivation Index,^14^ grouped into quintiles from 1 (least deprived) to 5 (most deprived).

#### Multimorbidity and polypharmacy

Long-term conditions (including diabetes) were identified from historical hospital and ProCare data using the 61 conditions included in the New Zealand M3 Multimorbidity Index^4^ (see also Supplementary Appendix S4). A condition was considered present if it had been recorded at least once before 1 January 2014. Multimorbidity was defined as ≥2 M3 conditions. Diagnostic information from primary care records was identified using Read codes, Read terms, and free text, processed using a previously described text-classification approach.^11^

Polypharmacy was defined as dispensing of five or more long-term medications during two or more consecutive 3-month periods, in accordance with the methodology of the New Zealand Health Quality and Safety Commission.^15^

### Outcome measures

The primary outcome was dispensing of dual therapy for primary prevention, defined as concurrent dispensing of blood pressure-lowering and lipid-lowering medications (Supplementary Appendix S5). Baseline dispensing was identified from dispensing records during the 6 months preceding the index date and categorised according to 5-year CVD risk: low (<5%), intermediate (5–14%), and high (≥15%).

Among participants receiving dual therapy at baseline, maintenance over the 2-year follow-up was assessed across four consecutive 6-month blocks, allowing for delayed prescription refills (although these medications are typically prescribed in 3-month intervals). Maintenance was defined as ≥75% coverage, corresponding to dispensing in at least three of the four follow-up blocks. Participants who developed CVD during follow-up were retained because sensitivity analyses excluding post-event follow-up did not materially change the results.

### Statistical analysis

Associations between multimorbidity and dual therapy were first examined using unadjusted Poisson regression models including an interaction between multimorbidity and CVD risk group. Multivariable Poisson regression models were then fitted within each CVD risk group to estimate adjusted relative risks (RRs). Covariates included multimorbidity, diabetes, sex, age group, prioritised ethnicity, and deprivation quintile. Multicollinearity was assessed using variance inflation factors (VIFs).

Among participants receiving dual therapy at baseline, maintenance was compared by multimorbidity status within each CVD risk group using χ^2^ tests. For patients who died during follow-up, blocks occurring after death were excluded from the denominator.

All analyses were performed using R version 4.5.0 (R Core Team, 2025).

## Results

### Cohort and baseline characteristics

**Figure 1.**
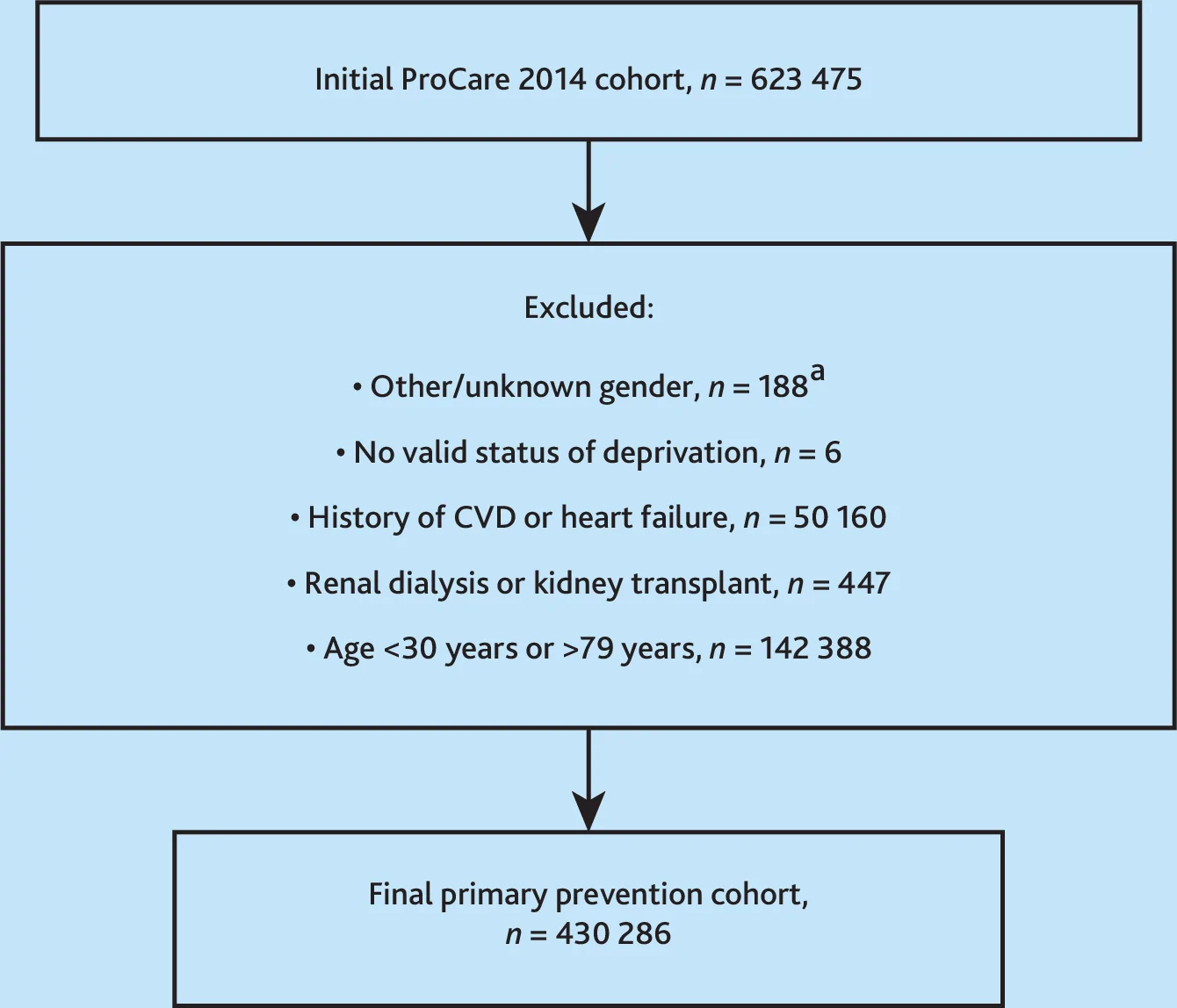
Flowchart of participant selection. ^a^Excluded individuals with sex recorded as other (*n* = 42) or unknown (*n* = 146). CVD = cardiovascular disease.

After applying exclusion criteria sequentially, 430 286 participants remained in the primary prevention cohort (Figure 1).

Table 1 shows the baseline characteristics of the cohort, stratified by CVD risk group. Most participants (81%) were classified as low risk, 16% as intermediate risk, and 2.3% as high risk. Overall, 21% had multimorbidity, with prevalence increasing across CVD risk groups from 16% in the low-risk group to 43% and 61% in the intermediate- and high-risk groups, respectively.

**Table 1.** Baseline participant characteristics, by 5-year CVD risk, categorised as low (<5%), intermediate (5–14%), and high (≥15%)

	CVD risk, *n* (%)			
	Low	Intermediate	High	Overall, *n* (%)
**Total**	350 453 (81)	69 862 (16)	9971 (2.3)	430 286 (100)
**Multimorbidity**	55 984 (16)	29 736 (43)	6073 (61)	91 793 (21)
**Age in years**				
30–59	317 396 (91)	18 557 (27)	689 (6.9)	336 642 (78)
60–69	31 317 (89)	30 786 (44)	2375 (24)	64 478 (15)
70–79	1740 (0.5)	20 519 (29)	6907 (69)	29 166 (6.8)
**Sex**				
Female	202 922 (58)	27 102 (39)	2655 (27)	232 679 (54)
Male	147 531 (42)	42 760 (61)	7316 (73)	197 607 (46)
**Diabetes**	12 365 (3.5)	18 517 (27)	4720 (47)	35 602 (8.3)
**Prioritised ethnicity**				
European	205 801 (59)	41 480 (59)	5863 (59)	253 144 (59)
Māori	26 541 (7.6)	7473 (11)	1550 (16)	35 564 (8.3)
Pacific peoples	29 853 (8.5)	9389 (13)	1388 (14)	40 630 (9.4)
Indian	26 607 (7.6)	5025 (7.2)	642 (6.4)	32 274 (7.5)
Chinese	29 101 (8.3)	2742 (3.9)	92 (0.9)	31 935 (7.4)
Other	32 550 (9.3)	3753 (5.4)	436 (4.4)	36 739 (8.5)
**New Zealand Deprivation Index quintile**				
Q1 (least deprivation)	94 104 (27)	15 102 (22)	1104 (11)	110 310 (26)
Q2	81 998 (23)	14 049 (20)	1456 (15)	97 503 (23)
Q3	65 307 (19)	12 626 (18)	1748 (18)	79 681 (19)
Q4	56 390 (16)	12 490 (18)	2265 (23)	71 145 (17)
Q5 (most deprivation)	52 654 (15)	15 595 (22)	3398 (34)	71 647 (17)
**Medications at baseline**				
BP lowering	27 927 (8.0)	37 970 (54)	7498 (75)	73 395 (17)
Lipid lowering^a^	21 468 (6.1)	27 255 (39)	4398 (44)	53 121 (12)
Dual therapy^a^	8741 (2.5)	21 543 (31)	3997 (40)	34 281 (8.0)
Polypharmacy^a^	11 636 (3.3)	18 177 (26)	4597 (46)	34 410 (8.0)
**Ever smoker**	49 326 (14)	15 619 (22)	4209 (42)	69 154 (16)

^a^BP-lowering medications included ACE inhibitors, ARBs, beta-blockers, calcium channel blockers, thiazide diuretics, and others. ^b^Lipid-lowering medications included statins and others (listed in Supplementary Appendix S5). ^c^Dual therapy refers to the combination of BP- and lipid-lowering medication. ^d^Polypharmacy was defined as the use of ≥5 long-term medications. ACE = angiotensin-converting enzyme. ARB = angiotensin II receptor blocker. BP = blood pressure. CVD = cardiovascular disease

Europeans comprised 59% of the cohort, followed by Pacific peoples (9.4%), Māori (8.3%), Indian (7.5%), Chinese (7.4%), and those categorised as Other (8.5%). In the low-risk group, 91% of participants were aged 30–59 years, whereas 69% of those in the high-risk group were aged 70–79 years. Men were increasingly represented with increasing CVD risk. Diabetes prevalence increased from 3.5% in the low-risk group to 47% in the high-risk group. A socioeconomic gradient was also evident: only 11% of participants in the high-risk group lived in the least deprived quintile, compared with 34% living in the most deprived quintile.

Baseline medication use reflected CVD risk. Dual therapy was received by 2.5% (*n* = 8741) of participants in the low-risk group, 31% (*n* = 21 543) in the intermediate-risk group, and 40% (*n* = 3997) in the high-risk group. Polypharmacy was present in 3.3% of participants in the low-risk group and 46% of those in the high-risk group. The proportion of participants recorded as ever smokers increased from 14% in the low-risk group to 42% in the high-risk group.

### Effect of multimorbidity on dual therapy, stratified by CVD risk

**Figure 2.**
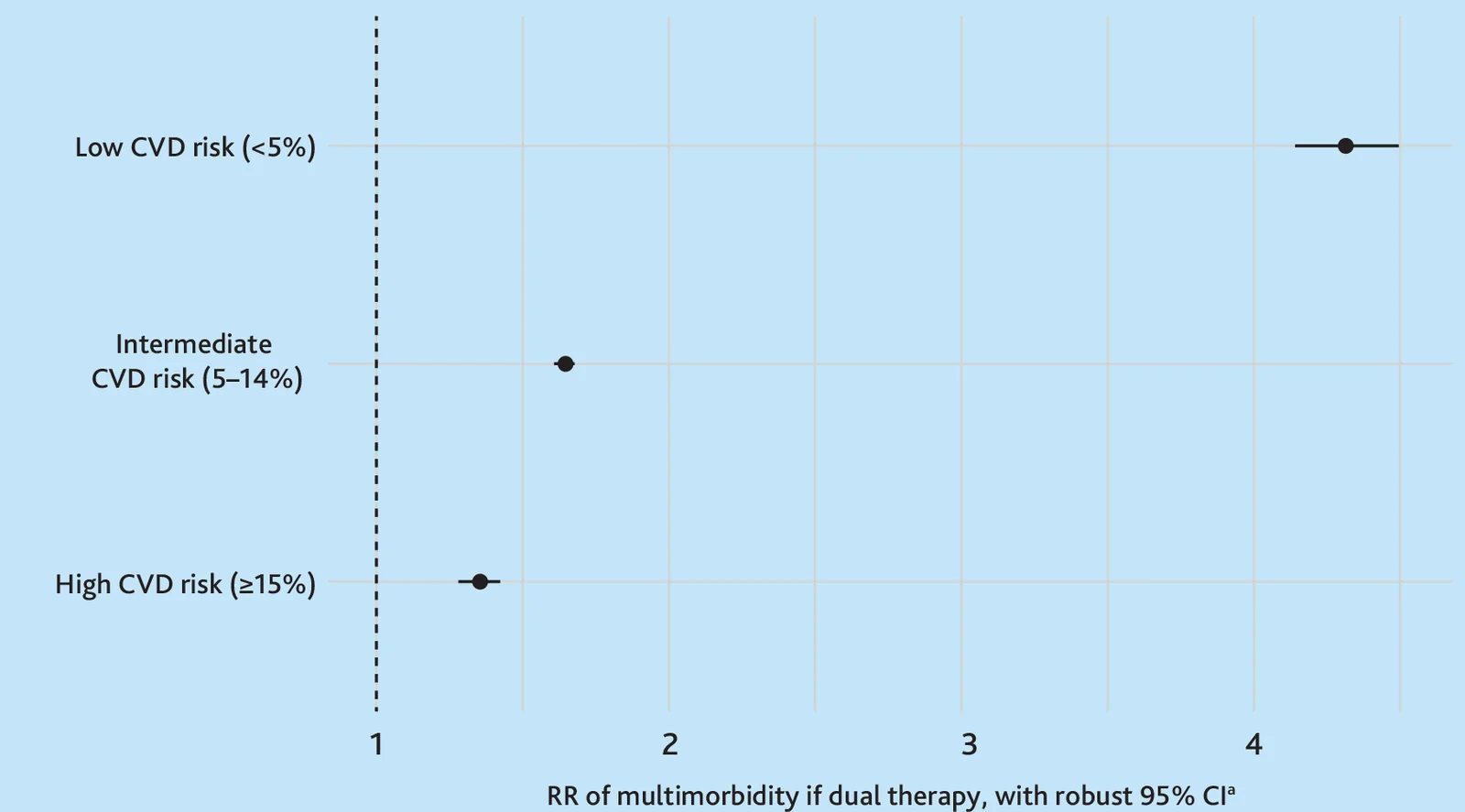
Association between multimorbidity and dual therapy across cardiovascular disese (CVD) risk groups ^a^Unadjusted relative risks (RRs) for dual therapy comparing participants with multimorbidity (≥2 long-term conditions [LTCs]) with those without multimorbidity (<2 LTCs), stratified by 5-year CVD risk (low <5%; intermediate 5–14%; high ≥15%). Estimates were derived from a Poisson regression model including an interaction between multimorbidity and CVD risk group. Within-stratum RRs (95% CIs) were: low risk, 4.32 (4.14–4.50); intermediate risk, 1.64 (1.61–1.68); and high risk, 1.35 (1.28–1.42) (all *P*<0.001).

Participants with multimorbidity were more likely to receive dual therapy across all CVD risk groups than those without. Unadjusted RRs for multimorbidity versus no multimorbidity were highest in the low-risk group (RR 4.32; 95% CI = 4.14 to 4.50), and attenuated for those at intermediate risk (RR 1.64; 95% CI = 1.61 to 1.68) and high risk (RR 1.35; 95% CI = 1.28 to 1.42) (Figure 2).

**Figure 3.**
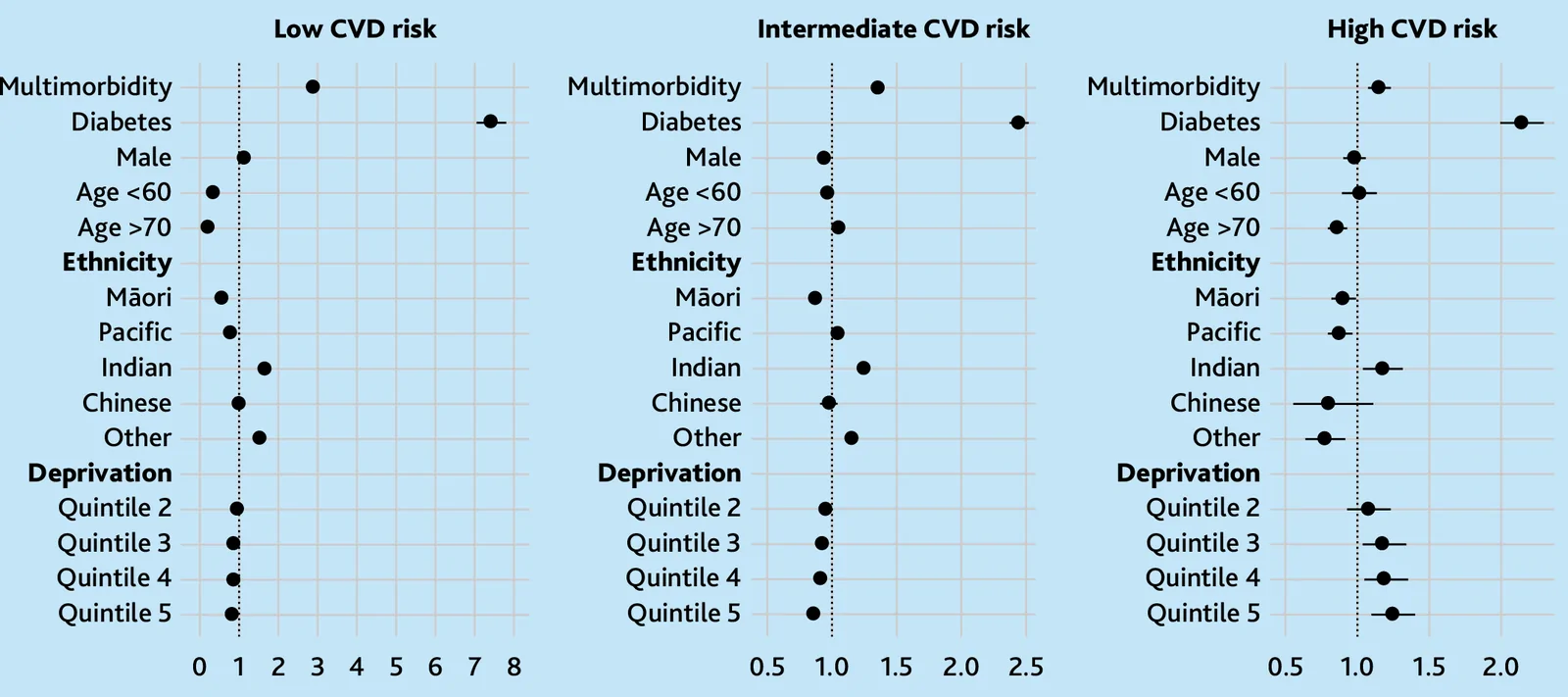
Multivariable associations between participant characteristics and dual therapy across cardiovascular disease (CVD) risk groups. ^a^Forest plots showing adjusted relative risks (RRs) and 95% confidence intervals (CIs) for receiving dual therapy at baseline, estimated using multivariable Poisson regression within five-year CVD risk groups: low (<5%), intermediate (5–14%), and high (≥15%) risk. Models included multimorbidity (reference: no multimorbidity), diabetes (reference: no diabetes), sex (reference: female), age group (reference: 60–69 years), prioritised ethnicity (reference: European), and neighbourhood deprivation (reference: New Zealand Deprivation Index quintile 1 [least deprived]). RRs >1 indicate a higher probability of receiving dual therapy relative to the reference category within each risk group. Adjusted RRs for multimorbidity were 2.90 (95% CI 2.77-3.03), 1.35 (95% CI 1.32-1.38), and 1.15 (95% CI 1.10 to 1.22) in the low-, intermediate-, and high-risk groups, respectively.

In multivariable models (Figure 3), multimorbidity and diabetes were consistently the strongest predictors of dual therapy across all CVD risk groups, with the strongest associations observed in the low-risk group (multimorbidity: RR 2.90, 95% CI = 2.77 to 3.03; diabetes: RR 7.45, 95% CI = 7.08-7.83). In the intermediate-risk group, the corresponding RRs were 1.35 (95% CI= 1.32 to 1.38) for multimorbidity and 2.46 (95% CI = 2.40 to 2.52) for diabetes. In the high-risk group, the corresponding RRs were 1.15 (95% CI = 1.10 to 1.22) and 2.15 (95% CI= 2.03 to 2.28), respectively.

Compared with Europeans, Māori people were less likely to receive dual therapy across all CVD risk groups, whereas people of Indian ethnicity were more likely to receive dual therapy. In the high-risk group, greater socioeconomic deprivation was associated with a higher likelihood of dual therapy.

Assessment of multicollinearity indicated low collinearity among model variables, with variance inflation factors consistently <2 across all models.

### Maintenance of dual therapy

**Figure 4.**
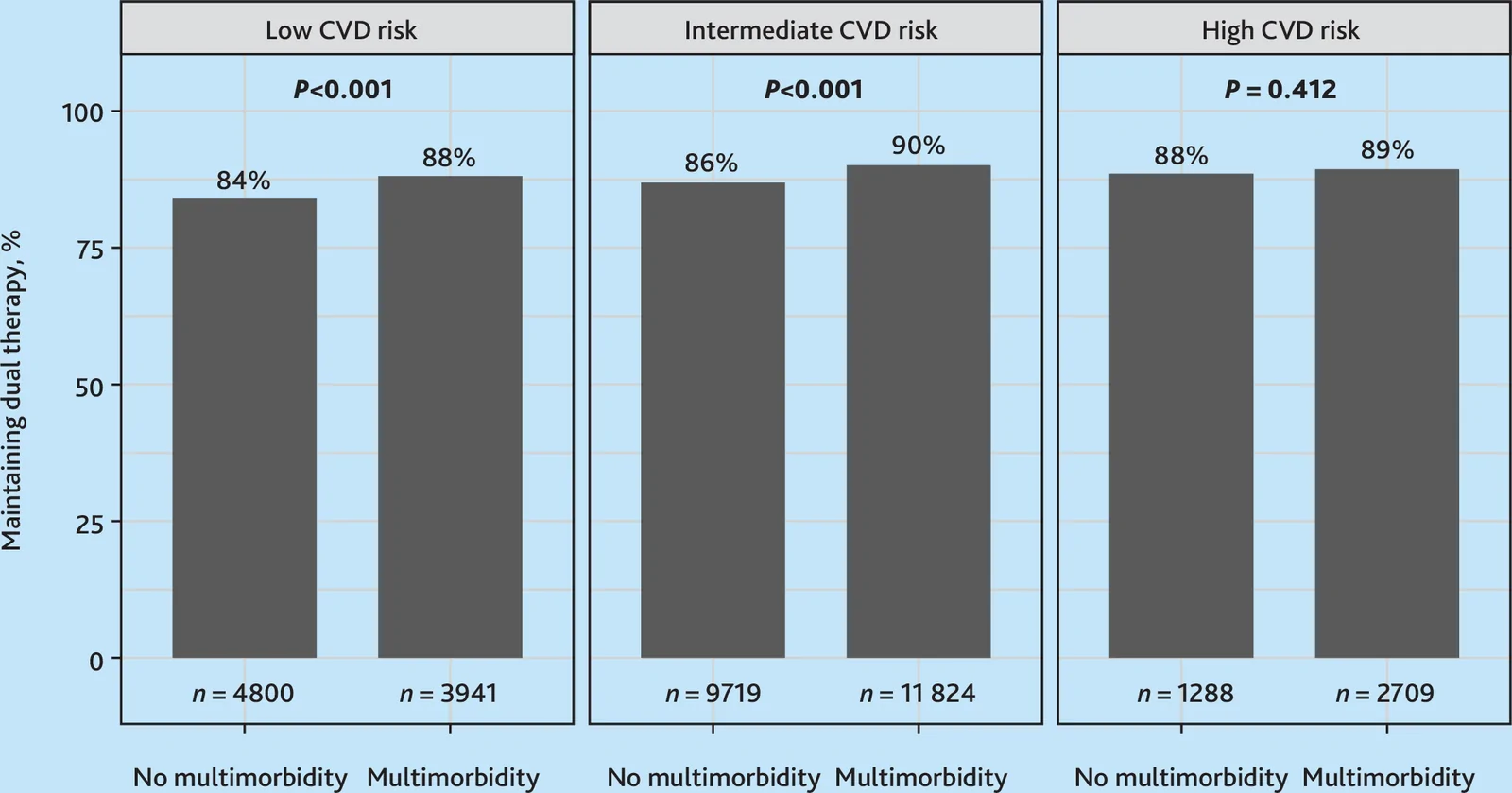
Maintenance of dual therapy according to multimorbidity across cardiovascular disease (CVD) risk groups. ^a^Bars represent the proportion of participants receiving dual therapy at baseline who maintained dual therapy (≥75% coverage over 2 years), stratified by 5-year CVD risk (low <5%; intermediate 5–14%; high ≥15%).

Data on maintenance of dual therapy are shown in Figure 4. Among participants receiving dual therapy at cohort entry, maintenance (≥75% coverage over 2 years) was high across all CVD risk groups, reaching at least 84%. In the high-risk group, maintenance was similarly high regardless of multimorbidity status (89% versus 88%; *P* = 0.412). In the low- and intermediate-risk groups, maintenance was slightly higher among participants with multimorbidity than among those without (Figure 4).

## Discussion

### Summary

In this large primary care cohort of people without prior CVD, dispensing of dual therapy increased with increasing CVD risk, consistent with New Zealand guideline recommendations. However, only 40% of people at high CVD risk received dual therapy, indicating a substantial evidence–practice gap. People with multimorbidity were more likely than those without multimorbidity to receive dual therapy across all CVD risk groups. Among those receiving dual therapy at baseline, people with multimorbidity in the low- and moderate-risk groups were also more likely to maintain therapy over 2 years, suggesting more sustained use of preventive treatment once initiated.

### Strengths and limitations

Key strengths include the large, population-based cohort, which provided sufficient statistical power for subgroup analyses, and the availability of complete data for the key study variables. In addition, the ethnic composition of the cohort was broadly representative of the New Zealand population.^16^ The use of routinely collected administrative health data is a validated and widely used approach in New Zealand population-based research, enabling complete capture of major cardiovascular events across the cohort.

One limitation is the potential under-ascertainment of diabetes managed without pharmacotherapy, as diabetes status in the risk equations relied on hospital data and medication dispensing. This may have resulted in residual confounding and overestimation of the association between multimorbidity and dual therapy, particularly among individuals classified as low CVD risk. Similarly, some individuals classified as being at low CVD risk by population-based risk equations may nevertheless have received dual therapy because their GPs considered them to be at higher risk based on individual clinical assessment. Such divergence may reflect appropriate clinical judgement, incorporating information not captured by risk equations, such as family history, longitudinal knowledge of the patient, or perceived vulnerability. Equally, it may reflect uncertainty in preventive decision making or overtreatment among some individuals classified as low risk. These findings highlight the importance of interpreting population-based risk estimates alongside clinical judgement when evaluating prescribing patterns. Conversely, the low prescribing rates observed among individuals at high estimated CVD risk may reflect a combination of clinician judgement, patient preferences, and system-level barriers, illustrating the complexity of translating guideline recommendations into clinical practice.

Ethnicity misclassification is another concern, given known quality issues in New Zealand health ethnicity data.^17^ Finally, medication dispensing contributed both to the CVD risk equations and to the study outcome, and several regression covariates were also components of the risk equations. Although this overlap could theoretically introduce collinearity, diagnostic testing indicated that its impact was minimal.

### Comparison with existing literature

Previous research has demonstrated that blood pressure- and lipid-lowering medications are more likely to be dispensed to people at higher CVD risk.^18,19^ However, our findings are consistent with previous reports of underuse of guideline-recommended dual therapy among people at high CVD risk,^8^ with the gap observed in the present study being even greater. Contrary to concerns that multimorbidity leads to poorer medication management,^20,21^ multimorbidity was associated with a higher likelihood of receiving and maintaining dual therapy. Diabetes was the strongest predictor of dual therapy within each CVD risk group, consistent with a French study reporting that multimorbidity including diabetes was associated with increased statin prescribing, whereas multimorbidity without diabetes was associated with lower statin prescribing.^22^ Maintenance was high overall and highest among people with multimorbidity at lower CVD risk, consistent with previous reports of better medication adherence among people with multimorbidity.^23^

Our findings also highlight socioeconomic and ethnic inequalities in guideline-based CVD prevention. Previous research has demonstrated that socioeconomic deprivation is associated with both a higher burden of CVD and inequities in preventive care.^4,24^ Among people at high estimated CVD risk, those living in more deprived areas were more likely to receive dual therapy, possibly reflecting recognition of their greater underlying risk. In contrast, among those classified as being at low or intermediate CVD risk, greater deprivation was associated with lower dispensing. This may indicate that socioeconomic deprivation is incorporated differently into prescribing decisions depending on CVD risk.

Māori, who experience a disproportionally high burden of CVD,^5^ were less likely to receive dual therapy, consistent with previous reports of inequities in cardiovascular risk assessment and management.^24^ A similar pattern was observed for Pacific peoples. In contrast, Indian people were more likely to receive dual therapy across all CVD risk groups, which may reflect the higher cardiovascular risk observed among Indian people in contemporary New Zealand CVD risk prediction equations.^6^ The lower likelihood of dual therapy among Māori and Pacific peoples is particularly concerning given the earlier onset and greater prevalence of multimorbidity^4^ as well as the reduced life expectancy observed in these populations compared with the overall New Zealand population.^25^

### Implications for research and practice

The results of the present study reinforce previous evidence highlighting the need for more proactive CVD risk assessment and management for Māori and Pacific peoples. These findings may also inform efforts in other countries to improve equitable implementation of guideline-based CVD prevention among underserved populations. Future research should explore the reasons for these inequalities, including clinical decision making, barriers to healthcare access or treatment initiation, and broader structural factors.

The substantial evidence–practice gap in dual therapy among people at high CVD risk has important implications for primary health organisations seeking to improve the quality of CVD prevention. Since these data were collected, ProCare has implemented quality-improvement initiatives that have reportedly increased dual-therapy uptake among people at high CVD risk by approximately 20% (unpublished personal communication with Dr Allan Moffitt, 1 December 2025).

Because maintenance was high once therapy had been initiated, the findings presented here suggest that the greatest opportunity for improving guideline-concordant care lies in earlier CVD risk assessment and initiation of preventive therapy, particularly for people at high CVD risk and populations with the greatest burden of CVD, including Māori, Pacific peoples, and socioeconomically deprived communities.

## Data

Data can be requested via data access proposals. ProCare data access requires agreement from the contributing provider and approval from the Multi-Region Ethics Committee.
